# Aqua­chlorido{2-[2-(cyclo­hexyl­carbamo­thioyl-κ*S*)hydrazinyl­idene-κ*N* ^1^]propano­ato(2−)}phenyl­tin(IV)

**DOI:** 10.1107/S1600536810031715

**Published:** 2010-08-18

**Authors:** Md. Abu Affan, Md. Abdus Salam, Ismail Jusoh, Seik Weng Ng, Edward R. T. Tiekink

**Affiliations:** a Faculty of Resource Science and Technology, Universiti Malaysia Sarawak, 94300 Kota Samarahan, Sarawak, Malaysia; b Department of Chemistry, University of Malaya, 50603 Kuala Lumpur, Malaysia

## Abstract

In the title organotin compound, [Sn(C_6_H_5_)(C_10_H_15_N_3_O_2_S)Cl(H_2_O)], the Sn atom is coordinated by the S, O, and imine N atoms of the dinegative tridentate ligand, a chloride ligand, the *ipso*-C atom of a phenyl ligand and by a water mol­ecule in a distorted octa­hedral coordination environment. Coordin­ated water mol­ecules link the organotin mol­ecules by forming O—H⋯O hydrogen bonds with both carbonyl and carboxyl­ate O atoms, leading to 12-membered {⋯OCO⋯HOH⋯}_2_ synthons. This results in the formation of supra­molecular chains along the *c* axis. The chains pack in the *ac* plane and stack along the *b* axis with links between layers afforded by N—H⋯Cl hydrogen bonds.

## Related literature

For background to the biological activity of tin/organotin compounds, see: Gielen & Tiekink (2005 ▶). For related studies on organotin compounds, see: Affan *et al.* (2009 ▶); Zukerman-Schpector *et al.* (2009 ▶); Affan *et al.* (2010 ▶).
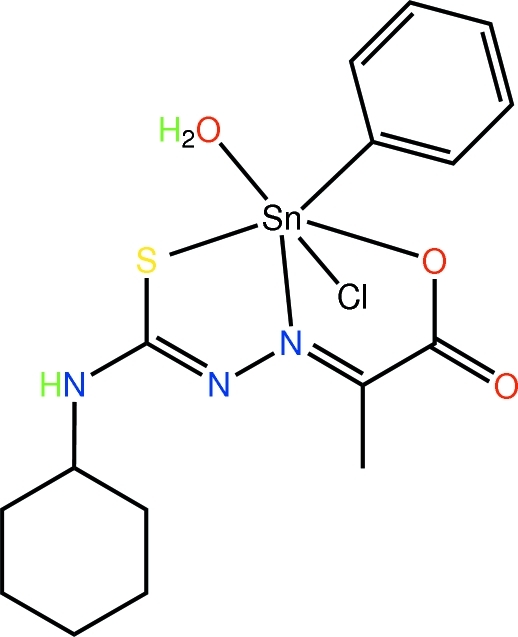


## Experimental

### 

#### Crystal data

[Sn(C_6_H_5_)(C_10_H_15_N_3_O_2_S)Cl(H_2_O)]
*M* *_r_* = 490.57
Monoclinic, 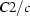

*a* = 16.3904 (9) Å
*b* = 19.2018 (10) Å
*c* = 13.1127 (7) Å
β = 108.4421 (7)°
*V* = 3915.0 (4) Å^3^
*Z* = 8
Mo *K*α radiation
μ = 1.57 mm^−1^
*T* = 100 K
0.30 × 0.25 × 0.20 mm

#### Data collection

Bruker SMART APEX diffractometer
Absorption correction: multi-scan (*SADABS*; Sheldrick, 1996 ▶) *T*_min_ = 0.613, *T*_max_ = 0.746
18020 measured reflections
4498 independent reflections
3853 reflections with *I* > 2σ(*I*)
*R*_int_ = 0.034

#### Refinement

*R*[*F* ^2^ > 2σ(*F* ^2^)] = 0.027
*wR*(*F* ^2^) = 0.091
*S* = 1.19
4498 reflections
239 parameters
3 restraints
H atoms treated by a mixture of independent and constrained refinement
Δρ_max_ = 0.54 e Å^−3^
Δρ_min_ = −0.53 e Å^−3^

### 

Data collection: *APEX2* (Bruker, 2009 ▶); cell refinement: *SAINT* (Bruker, 2009 ▶); data reduction: *SAINT*; program(s) used to solve structure: *SHELXS97* (Sheldrick, 2008 ▶); program(s) used to refine structure: *SHELXL97* (Sheldrick, 2008 ▶); molecular graphics: *ORTEP-3* (Farrugia, 1997 ▶) and *DIAMOND* (Brandenburg, 2006 ▶); software used to prepare material for publication: *publCIF* (Westrip, 2010 ▶).

## Supplementary Material

Crystal structure: contains datablocks global, I. DOI: 10.1107/S1600536810031715/lh5114sup1.cif

Structure factors: contains datablocks I. DOI: 10.1107/S1600536810031715/lh5114Isup2.hkl

Additional supplementary materials:  crystallographic information; 3D view; checkCIF report

## Figures and Tables

**Table 1:** Selected bond lengths (Å)

Sn—C11	2.123 (3)
Sn—O1	2.148 (2)
Sn—N3	2.195 (3)
Sn—O1w	2.224 (2)
Sn—Cl1	2.4524 (8)
Sn—S1	2.4598 (7)

**Table 2:** Hydrogen-bond geometry (Å, °)

*D*—H⋯*A*	*D*—H	H⋯*A*	*D*⋯*A*	*D*—H⋯*A*
O1w—H1*w*⋯O1^i^	0.84 (5)	1.94 (3)	2.733 (3)	159 (6)
O1*w*—H2w⋯O2^ii^	0.83 (5)	1.81 (2)	2.645 (3)	174 (5)
N1—H1*n*⋯Cl1^iii^	0.86 (3)	2.59 (2)	3.407 (3)	161 (3)

Symmetry codes: (i) 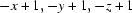; (ii) 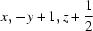; (iii) 
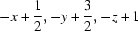
.

**Fig. 1.**
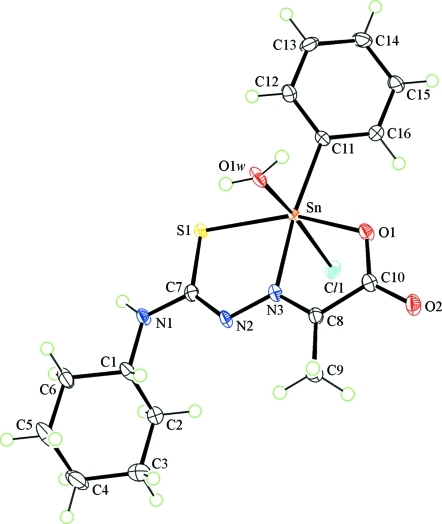
The molecular structure of (I) showing the atom-labelling scheme and displacement ellipsoids at the 50% probability level.

**Fig. 2.**
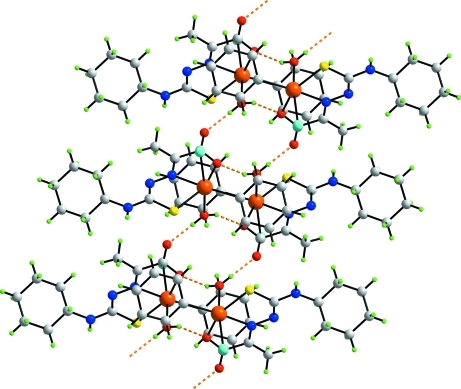
Supramolecular chains along c in the structure of (I). The O–H···O hydrogen bonds are shown as orange dashed lines.

**Fig. 3.**
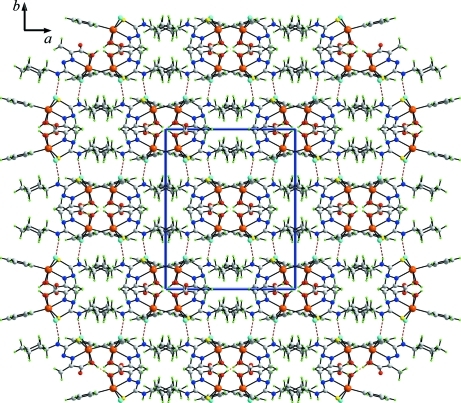
Unit-cell contents shown in projection down the c axis in (I). The N–H···Cl hydrogen bonds between layers are shown as brown dashed lines.

## supplementary crystallographic information

### Comment

Organotin compounds continue to attract considerable owing to the wide variety of biological properties (Gielen & Tiekink, 2005). In continuation of our work in this area (Affan *et al.*, 2009; Zukerman-Schpector *et al.*, 2009; Affan *et al.* 2010), the title organotin compound, (I), was synthesized and structurally characterized.

The Sn atom is coordinated *via* the S, O, and imine-N atoms of the dinegative tridentate ligand, thereby forming two planar five-membered chelate rings. The distorted CC*l*NO_2_S octahedral coordination geometry is completed by an aqua ligand, a chloride atom, and the *ipso*-C atom of the phenyl group, Table 1. The greatest distortion from the ideal octahedral geometry is found in the O1–Sn–S1 angle of 153.73 (6) °, a feature which arises due to the restricted bite distances of the chelate rings.

The most notable feature of the crystal packing is the formation of O–H···O and N–H···Cl hydrogen bonds, Table 1. The water molecule hydrogen bonds to a carbonyl-O of one molecule and a carboxyl-O of another. Two-fold symmetry leads to the formation of a 12-membered {···OCO···HOH···}_2_ synthon and the formation of a supramolecular chain along the *c* axis, Fig. 2. The chains pack in the *ac* plane and stack along the *b* axis with the primary interactions between successive layers being hydrogen bonds of the type N–H···Cl, Fig. 3.

### Experimental

The pyruvic acid cyclohexyl thiosemicarbazone ligand (0.243 g, 1.0 mmol) was dissolved in dry methanol (10 ml) in a Schlenk apparatus under a purified dry nitrogen atmosphere. Phenyltin(IV) trichloride (0.302 g, 1.0 mmol) dissolved in absolute methanol (10 ml) was added drop-wise. The resulting mixture was refluxed for 5 h. The resulting solid was filtered and dried *in vacuo* over silica gel. Re-crystallization was by slow evaporation of its methanol solution yielded light-brown crystals of (I). Yield 0.43 g, 78%: *M*.pt.: 477–479 K. Anal. Calc. for C_16_H_22_ClN_3_O_3_SSn: C, 39.17; H, 4.52; N, 8.56%. Found: C, 39.16; H, 4.50; N, 8.54%

### Refinement

Carbon-bound H-atoms were placed in calculated positions (C—H 0.95 to 1.00 Å) and were included in the refinement in the riding model approximation, with *U*_iso_(H) set to 1.2 to 1.5*U*_eq_(C). The O– and N-bound H-atoms were located in a difference Fourier map, and was refined with distance restraints of O–H = 0.84 ±0.01 Å and N–H = 0.86±0.01 Å; the *U_iso_* values were freely refined

### Crystal data

**Table:**

[Sn(C_6_H_5_)(C_10_H_15_N_3_O_2_S)Cl(H_2_O)]	*F*(000) = 1968
*M**_r_* = 490.57	*D*_x_ = 1.665 Mg m^−^^3^
Monoclinic, *C*2/*c*	Mo *K*α radiation, λ = 0.71073 Å
Hall symbol: -C 2yc	Cell parameters from 8808 reflections
*a* = 16.3904 (9) Å	θ = 2.6–28.3°
*b* = 19.2018 (10) Å	µ = 1.57 mm^−^^1^
*c* = 13.1127 (7) Å	*T* = 100 K
β = 108.4421 (7)°	Block, light-brown
*V* = 3915.0 (4) Å^3^	0.30 × 0.25 × 0.20 mm
*Z* = 8	

### Data collection

**Table:**

Bruker SMART APEX diffractometer	4498 independent reflections
Radiation source: fine-focus sealed tube	3853 reflections with *I* > 2σ(*I*)
graphite	*R*_int_ = 0.034
ω scan	θ_max_ = 27.5°, θ_min_ = 1.7°
Absorption correction: multi-scan (*SADABS*; Sheldrick, 1996)	*h* = −21→21
*T*_min_ = 0.613, *T*_max_ = 0.746	*k* = −24→24
18020 measured reflections	*l* = −16→17

### Refinement

**Table:**

Refinement on *F*^2^	Primary atom site location: structure-invariant direct methods
Least-squares matrix: full	Secondary atom site location: difference Fourier map
*R*[*F*^2^ > 2σ(*F*^2^)] = 0.027	Hydrogen site location: inferred from neighbouring sites
*wR*(*F*^2^) = 0.091	H atoms treated by a mixture of independent and constrained refinement
*S* = 1.19	*w* = 1/[σ^2^(*F*_o_^2^) + (0.05*P*)^2^ + 0.5*P*] where *P* = (*F*_o_^2^ + 2*F*_c_^2^)/3
4498 reflections	(Δ/σ)_max_ = 0.001
239 parameters	Δρ_max_ = 0.54 e Å^−^^3^
3 restraints	Δρ_min_ = −0.53 e Å^−^^3^

### Special details

**Table:**

Geometry. All s.u.'s (except the s.u. in the dihedral angle between two l.s. planes) are estimated using the full covariance matrix. The cell s.u.'s are taken into account individually in the estimation of s.u.'s in distances, angles and torsion angles; correlations between s.u.'s in cell parameters are only used when they are defined by crystal symmetry. An approximate (isotropic) treatment of cell s.u.'s is used for estimating s.u.'s involving l.s. planes.
Refinement. Refinement of *F*^2^ against ALL reflections. The weighted *R*-factor *wR* and goodness of fit *S* are based on *F*^2^, conventional *R*-factors *R* are based on *F*, with *F* set to zero for negative *F*^2^. The threshold expression of *F*^2^ > 2σ(*F*^2^) is used only for calculating *R*-factors(gt) *etc*. and is not relevant to the choice of reflections for refinement. *R*-factors based on *F*^2^ are statistically about twice as large as those based on *F*, and *R*- factors based on ALL data will be even larger.

### Fractional atomic coordinates and isotropic or equivalent isotropic displacement parameters (Å^2^)

**Table:**

	*x*	*y*	*z*	*U*_iso_*/*U*_eq_	
Sn	0.404486 (12)	0.620716 (10)	0.467538 (16)	0.01146 (8)	
Cl1	0.34899 (5)	0.69580 (4)	0.30936 (6)	0.01767 (16)	
S1	0.32710 (5)	0.68215 (4)	0.57359 (6)	0.01380 (16)	
O1	0.41904 (13)	0.54024 (11)	0.36085 (18)	0.0163 (5)	
O2	0.34146 (15)	0.47501 (12)	0.22370 (19)	0.0209 (5)	
O1W	0.43443 (15)	0.53800 (13)	0.5926 (2)	0.0201 (5)	
H1W	0.475 (3)	0.512 (3)	0.590 (5)	0.09 (2)*	
H2W	0.406 (3)	0.531 (3)	0.634 (3)	0.066 (17)*	
N1	0.16705 (17)	0.65474 (14)	0.5583 (2)	0.0158 (5)	
H1N	0.176 (2)	0.6892 (13)	0.602 (2)	0.019 (10)*	
N2	0.21381 (16)	0.58361 (13)	0.4501 (2)	0.0151 (5)	
N3	0.27999 (16)	0.56696 (13)	0.4134 (2)	0.0140 (5)	
C1	0.0801 (2)	0.62496 (16)	0.5200 (3)	0.0182 (7)	
H1A	0.0852	0.5735	0.5119	0.022*	
C2	0.0277 (2)	0.6546 (2)	0.4129 (3)	0.0275 (8)	
H2A	0.0576	0.6459	0.3593	0.033*	
H2B	0.0219	0.7056	0.4195	0.033*	
C3	−0.0616 (3)	0.6211 (3)	0.3750 (4)	0.0455 (12)	
H3A	−0.0958	0.6422	0.3058	0.055*	
H3B	−0.0558	0.5707	0.3630	0.055*	
C4	−0.1082 (2)	0.6313 (2)	0.4576 (4)	0.0354 (10)	
H4A	−0.1207	0.6815	0.4625	0.042*	
H4B	−0.1637	0.6061	0.4339	0.042*	
C5	−0.0553 (2)	0.6053 (2)	0.5669 (4)	0.0324 (9)	
H5A	−0.0496	0.5540	0.5644	0.039*	
H5B	−0.0852	0.6163	0.6197	0.039*	
C6	0.0344 (2)	0.63821 (19)	0.6035 (3)	0.0230 (7)	
H6A	0.0294	0.6890	0.6133	0.028*	
H6B	0.0687	0.6181	0.6735	0.028*	
C7	0.2306 (2)	0.63535 (16)	0.5207 (3)	0.0140 (6)	
C8	0.27078 (19)	0.52131 (16)	0.3388 (2)	0.0147 (6)	
C9	0.1909 (2)	0.48168 (17)	0.2862 (3)	0.0204 (7)	
H9A	0.1428	0.5030	0.3041	0.031*	
H9B	0.1982	0.4334	0.3116	0.031*	
H9C	0.1789	0.4825	0.2081	0.031*	
C10	0.34849 (19)	0.51063 (16)	0.3033 (3)	0.0155 (6)	
C11	0.53637 (18)	0.64917 (15)	0.5141 (2)	0.0131 (6)	
C12	0.5831 (2)	0.66164 (17)	0.6212 (3)	0.0198 (7)	
H12A	0.5558	0.6584	0.6750	0.024*	
C13	0.6698 (2)	0.67889 (19)	0.6493 (3)	0.0226 (7)	
H13	0.7013	0.6884	0.7222	0.027*	
C14	0.7103 (2)	0.68218 (19)	0.5720 (3)	0.0230 (7)	
H14	0.7697	0.6935	0.5919	0.028*	
C15	0.6644 (2)	0.66898 (18)	0.4644 (3)	0.0221 (7)	
H15	0.6923	0.6708	0.4112	0.027*	
C16	0.5775 (2)	0.65323 (17)	0.4362 (3)	0.0187 (7)	
H16	0.5457	0.6451	0.3629	0.022*	

### Atomic displacement parameters (Å^2^)

**Table:**

	*U*^11^	*U*^22^	*U*^33^	*U*^12^	*U*^13^	*U*^23^
Sn	0.01020 (12)	0.01204 (12)	0.01312 (13)	0.00088 (7)	0.00509 (8)	−0.00039 (7)
Cl1	0.0226 (4)	0.0173 (4)	0.0131 (4)	0.0036 (3)	0.0056 (3)	0.0019 (3)
S1	0.0125 (3)	0.0155 (4)	0.0143 (4)	0.0011 (3)	0.0054 (3)	−0.0021 (3)
O1	0.0140 (10)	0.0154 (11)	0.0216 (12)	−0.0001 (8)	0.0087 (9)	−0.0032 (9)
O2	0.0226 (12)	0.0207 (12)	0.0239 (13)	−0.0031 (9)	0.0137 (10)	−0.0074 (10)
O1W	0.0162 (11)	0.0218 (12)	0.0270 (13)	0.0088 (9)	0.0137 (10)	0.0105 (10)
N1	0.0133 (12)	0.0166 (14)	0.0200 (14)	0.0002 (10)	0.0089 (11)	−0.0040 (11)
N2	0.0128 (12)	0.0174 (13)	0.0187 (14)	0.0013 (10)	0.0099 (10)	−0.0015 (11)
N3	0.0147 (12)	0.0135 (12)	0.0163 (13)	0.0017 (10)	0.0083 (10)	0.0009 (10)
C1	0.0138 (15)	0.0158 (16)	0.0284 (19)	−0.0014 (11)	0.0117 (14)	−0.0014 (13)
C2	0.0185 (17)	0.045 (2)	0.0203 (18)	−0.0013 (15)	0.0084 (14)	−0.0053 (16)
C3	0.021 (2)	0.078 (4)	0.035 (3)	−0.0079 (19)	0.0054 (17)	−0.024 (2)
C4	0.0161 (17)	0.045 (3)	0.048 (3)	−0.0062 (16)	0.0146 (17)	−0.0146 (19)
C5	0.0234 (19)	0.0272 (19)	0.056 (3)	−0.0001 (15)	0.0258 (19)	0.0030 (18)
C6	0.0174 (16)	0.0292 (18)	0.0270 (19)	0.0058 (14)	0.0136 (14)	0.0069 (15)
C7	0.0163 (15)	0.0127 (14)	0.0148 (15)	0.0012 (11)	0.0073 (12)	0.0023 (12)
C8	0.0147 (14)	0.0155 (15)	0.0151 (15)	0.0018 (11)	0.0066 (12)	−0.0004 (12)
C9	0.0184 (16)	0.0188 (17)	0.0260 (18)	−0.0035 (13)	0.0100 (14)	−0.0066 (14)
C10	0.0165 (15)	0.0137 (15)	0.0184 (16)	0.0006 (12)	0.0085 (12)	0.0009 (12)
C11	0.0113 (13)	0.0123 (14)	0.0164 (15)	0.0010 (11)	0.0056 (11)	0.0023 (12)
C12	0.0166 (15)	0.0265 (18)	0.0186 (17)	−0.0005 (13)	0.0090 (13)	0.0020 (14)
C13	0.0179 (16)	0.034 (2)	0.0126 (16)	−0.0060 (14)	−0.0001 (13)	0.0006 (14)
C14	0.0123 (15)	0.0324 (19)	0.0227 (18)	−0.0044 (13)	0.0034 (13)	0.0030 (15)
C15	0.0147 (15)	0.0342 (19)	0.0198 (17)	−0.0017 (14)	0.0088 (13)	0.0030 (15)
C16	0.0162 (15)	0.0255 (18)	0.0138 (16)	0.0002 (13)	0.0042 (12)	−0.0013 (13)

### Geometric parameters (Å, °)

**Table:**

Sn—C11	2.123 (3)	C3—H3B	0.9900
Sn—O1	2.148 (2)	C4—C5	1.506 (6)
Sn—N3	2.195 (3)	C4—H4A	0.9900
Sn—O1w	2.224 (2)	C4—H4B	0.9900
Sn—Cl1	2.4524 (8)	C5—C6	1.532 (5)
Sn—S1	2.4598 (7)	C5—H5A	0.9900
S1—C7	1.759 (3)	C5—H5B	0.9900
O1—C10	1.295 (4)	C6—H6A	0.9900
O2—C10	1.223 (4)	C6—H6B	0.9900
O1W—H1W	0.84 (5)	C8—C9	1.482 (4)
O1W—H2W	0.83 (5)	C8—C10	1.502 (4)
N1—C7	1.339 (4)	C9—H9A	0.9800
N1—C1	1.469 (4)	C9—H9B	0.9800
N1—H1N	0.86 (3)	C9—H9C	0.9800
N2—C7	1.326 (4)	C11—C12	1.391 (4)
N2—N3	1.357 (3)	C11—C16	1.392 (4)
N3—C8	1.286 (4)	C12—C13	1.390 (4)
C1—C2	1.507 (5)	C12—H12A	0.9500
C1—C6	1.530 (4)	C13—C14	1.377 (5)
C1—H1A	1.0000	C13—H13	0.9500
C2—C3	1.531 (5)	C14—C15	1.395 (5)
C2—H2A	0.9900	C14—H14	0.9500
C2—H2B	0.9900	C15—C16	1.387 (4)
C3—C4	1.524 (6)	C15—H15	0.9500
C3—H3A	0.9900	C16—H16	0.9500
			
C11—Sn—O1	93.42 (10)	C5—C4—H4B	109.3
C11—Sn—N3	166.35 (10)	C3—C4—H4B	109.3
O1—Sn—N3	74.60 (9)	H4A—C4—H4B	108.0
C11—Sn—O1W	90.27 (10)	C4—C5—C6	111.6 (3)
O1—Sn—O1W	85.55 (9)	C4—C5—H5A	109.3
N3—Sn—O1W	82.43 (9)	C6—C5—H5A	109.3
C11—Sn—Cl1	99.40 (8)	C4—C5—H5B	109.3
O1—Sn—Cl1	87.71 (6)	C6—C5—H5B	109.3
N3—Sn—Cl1	86.84 (7)	H5A—C5—H5B	108.0
O1W—Sn—Cl1	168.53 (7)	C1—C6—C5	110.2 (3)
C11—Sn—S1	111.98 (8)	C1—C6—H6A	109.6
O1—Sn—S1	153.73 (6)	C5—C6—H6A	109.6
N3—Sn—S1	79.37 (7)	C1—C6—H6B	109.6
O1W—Sn—S1	87.63 (6)	C5—C6—H6B	109.6
Cl1—Sn—S1	94.39 (3)	H6A—C6—H6B	108.1
C7—S1—Sn	95.30 (10)	N2—C7—N1	116.8 (3)
C10—O1—Sn	115.62 (18)	N2—C7—S1	128.3 (2)
Sn—O1W—H1W	113 (4)	N1—C7—S1	114.8 (2)
Sn—O1W—H2W	124 (4)	N3—C8—C9	125.3 (3)
H1W—O1W—H2W	123 (5)	N3—C8—C10	114.9 (3)
C7—N1—C1	123.4 (3)	C9—C8—C10	119.8 (3)
C7—N1—H1N	119 (3)	C8—C9—H9A	109.5
C1—N1—H1N	118 (2)	C8—C9—H9B	109.5
C7—N2—N3	114.3 (2)	H9A—C9—H9B	109.5
C8—N3—N2	121.0 (3)	C8—C9—H9C	109.5
C8—N3—Sn	116.1 (2)	H9A—C9—H9C	109.5
N2—N3—Sn	122.70 (19)	H9B—C9—H9C	109.5
N1—C1—C2	112.1 (3)	O2—C10—O1	124.6 (3)
N1—C1—C6	109.4 (3)	O2—C10—C8	118.7 (3)
C2—C1—C6	109.9 (3)	O1—C10—C8	116.6 (3)
N1—C1—H1A	108.4	C12—C11—C16	119.4 (3)
C2—C1—H1A	108.4	C12—C11—Sn	121.3 (2)
C6—C1—H1A	108.4	C16—C11—Sn	119.2 (2)
C1—C2—C3	110.4 (3)	C13—C12—C11	119.9 (3)
C1—C2—H2A	109.6	C13—C12—H12A	120.0
C3—C2—H2A	109.6	C11—C12—H12A	120.0
C1—C2—H2B	109.6	C14—C13—C12	120.4 (3)
C3—C2—H2B	109.6	C14—C13—H13	119.8
H2A—C2—H2B	108.1	C12—C13—H13	119.8
C4—C3—C2	111.0 (3)	C13—C14—C15	120.3 (3)
C4—C3—H3A	109.4	C13—C14—H14	119.9
C2—C3—H3A	109.4	C15—C14—H14	119.9
C4—C3—H3B	109.4	C16—C15—C14	119.3 (3)
C2—C3—H3B	109.4	C16—C15—H15	120.3
H3A—C3—H3B	108.0	C14—C15—H15	120.3
C5—C4—C3	111.5 (3)	C15—C16—C11	120.7 (3)
C5—C4—H4A	109.3	C15—C16—H16	119.7
C3—C4—H4A	109.3	C11—C16—H16	119.7
			
C11—Sn—S1—C7	−173.02 (13)	N3—N2—C7—S1	−1.7 (4)
O1—Sn—S1—C7	−8.72 (18)	C1—N1—C7—N2	−4.2 (5)
N3—Sn—S1—C7	−0.95 (12)	C1—N1—C7—S1	176.4 (2)
O1W—Sn—S1—C7	−83.69 (12)	Sn—S1—C7—N2	1.9 (3)
Cl1—Sn—S1—C7	85.00 (10)	Sn—S1—C7—N1	−178.8 (2)
C11—Sn—O1—C10	−173.8 (2)	N2—N3—C8—C9	−0.9 (5)
N3—Sn—O1—C10	12.9 (2)	Sn—N3—C8—C9	−176.5 (2)
O1W—Sn—O1—C10	96.2 (2)	N2—N3—C8—C10	177.5 (3)
Cl1—Sn—O1—C10	−74.5 (2)	Sn—N3—C8—C10	1.9 (3)
S1—Sn—O1—C10	20.8 (3)	Sn—O1—C10—O2	164.0 (3)
C7—N2—N3—C8	−174.9 (3)	Sn—O1—C10—C8	−16.3 (3)
C7—N2—N3—Sn	0.4 (4)	N3—C8—C10—O2	−170.7 (3)
C11—Sn—N3—C8	−36.8 (6)	C9—C8—C10—O2	7.9 (5)
O1—Sn—N3—C8	−7.6 (2)	N3—C8—C10—O1	9.6 (4)
O1W—Sn—N3—C8	−95.0 (2)	C9—C8—C10—O1	−171.9 (3)
Cl1—Sn—N3—C8	80.9 (2)	O1—Sn—C11—C12	−135.8 (3)
S1—Sn—N3—C8	176.0 (2)	N3—Sn—C11—C12	−107.6 (5)
C11—Sn—N3—N2	147.7 (4)	O1W—Sn—C11—C12	−50.2 (3)
O1—Sn—N3—N2	176.9 (2)	Cl1—Sn—C11—C12	136.0 (2)
O1W—Sn—N3—N2	89.5 (2)	S1—Sn—C11—C12	37.3 (3)
Cl1—Sn—N3—N2	−94.6 (2)	O1—Sn—C11—C16	42.1 (3)
S1—Sn—N3—N2	0.5 (2)	N3—Sn—C11—C16	70.3 (5)
C7—N1—C1—C2	−77.7 (4)	O1W—Sn—C11—C16	127.7 (3)
C7—N1—C1—C6	160.1 (3)	Cl1—Sn—C11—C16	−46.1 (3)
N1—C1—C2—C3	178.8 (3)	S1—Sn—C11—C16	−144.8 (2)
C6—C1—C2—C3	−59.3 (4)	C16—C11—C12—C13	0.9 (5)
C1—C2—C3—C4	57.3 (5)	Sn—C11—C12—C13	178.8 (3)
C2—C3—C4—C5	−54.5 (5)	C11—C12—C13—C14	−1.5 (5)
C3—C4—C5—C6	54.2 (5)	C12—C13—C14—C15	0.7 (6)
N1—C1—C6—C5	−178.0 (3)	C13—C14—C15—C16	0.7 (5)
C2—C1—C6—C5	58.5 (4)	C14—C15—C16—C11	−1.3 (5)
C4—C5—C6—C1	−56.1 (4)	C12—C11—C16—C15	0.5 (5)
N3—N2—C7—N1	178.9 (3)	Sn—C11—C16—C15	−177.5 (3)

### Hydrogen-bond geometry (Å, °)

**Table:**

*D*—H···*A*	*D*—H	H···*A*	*D*···*A*	*D*—H···*A*
O1w—H1w···O1^i^	0.84 (5)	1.94 (3)	2.733 (3)	159 (6)
O1w—H2w···O2^ii^	0.83 (5)	1.81 (2)	2.645 (3)	174 (5)
N1—H1n···Cl1^iii^	0.86 (3)	2.587 (16)	3.407 (3)	161 (3)

Symmetry codes: (i) −*x*+1, −*y*+1, −*z*+1; (ii) *x*, −*y*+1, *z*+1/2; (iii) −*x*+1/2, −*y*+3/2, −*z*+1.
